# Supplementary material for: Exciton Manifolds in Highly Ambipolar Doped WS2
Source: Nanomaterials (Basel). 2022 Sep 19;12(18):3255. doi: 10.3390/nano12183255 (PMC9504948; doi:10.3390/nano12183255)
Supplement: Supplementary file 1 [file nanomaterials-12-03255-s001.zip › nanomaterials-1893219-supplementary.pdf]

Supplementary Materials: Exciton Manifolds in Highly Ambipolar Doped WS_2

David Otto Tiede [1,2], Nihit Saigal [1,2], Hossein Ostovar [1,2], Vera Döring [1,2], Hendrik Lambers[1,2], and Ursula Wurstbauer [1,2,*]

1. Photoluminescence Measurements

The PL measurements on devices with PE always presented a background fluorescence that is assigned to emission from the PE layer. Since this PE features a weak fluorescent signal in the relevant spectral range as confirmed by focusing the laser spot on a position on the samples without WS_2 but with PE, this background needs to be subtracted from the PL spectra from gated WS_2 for a quantitative analysis.

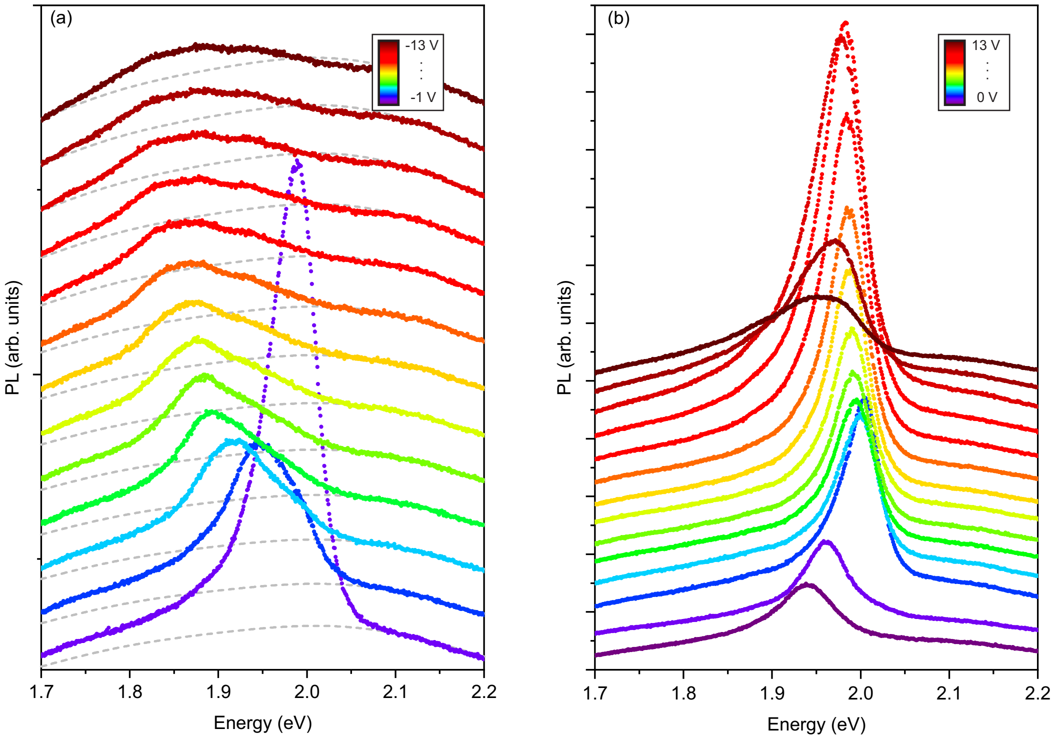

Figure S1. Raw gate voltage dependent PL spectra. (a) Negative voltage regime. Grey dotted lines indicates substracted background emission assigned to PE fluorescence. It was found that the PE PL emission can be described with a two Gaussian fit. The overall intensity of background emission changes slightly with the applied gate voltage, but does not change in shape. The obtained background emission thus is only intensity adjusted when applied to spectra with lower WS_2 emission, where the intensity correction factor varies between 0.8 and 1.2 (b) Positive voltage regime.

The background fluorescence intensity can change slightly with the applied gate voltage, but does not change in shape. The spectra are thus corrected via subtracting an intensity adapted, shape invariant PE background, where the background intensity slightly changes with the applied gate voltage. The error of the different peaks depends both on the background correction and the peak shape of the main emission peak. The background correction becomes more important in weak emission regimes (negative voltages and high positive voltages). Due to the two step fitting approach and the different fitting ranges, it is difficult to quantify the accumulative uncertainty for every fit. It is also affected by the number of assumed Gaussian peaks and the overall emission intensity of the WS_2 ML. In the high doping range resulting in a very weak WS_2 PL signal, the uncertainty of the

background correction becomes more important. Qualitatively speaking, a higher peak intensity and a low number of distinct Gaussian peaks reduce the uncertainty of the fit. In the highly negative doped regime, the low emission intensity is compensated by a lower number of Gaussian peaks, such that we estimate the uncertainty of the peak position to be reasonably low (estimated to be well below 5%).

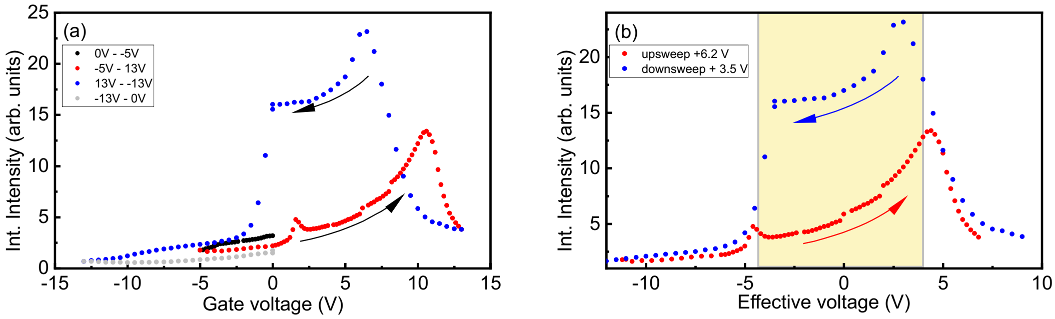

Figure S2. Hysteresis in gate voltage dependent measurements. (a) Integrated PL intensity in in dependence of gate voltage. Black arrows indicate sweep direction. (b) Hysteresis corrected PL intensity. The upsweep and downsweep curves are shifted such that an effective voltage of 0V corresponds to an expected Fermi level $E_F = 0$.

1.1. Low Temperature Photoluminescence Measurements

Low temperature measurements were carried out to obtain further information for the interpretation of the PL multiplet observed at room temperature. At cryogenic temperatures, PL1 and also an blueshifted PL line at the expected energy of A_{1s} occurs. The same energy difference as at the room temperature measurements as well as the nearly identical temperature dependent evolution of both peaks position corroborate the interpretation that both lines are due to interband transition between electrons at the spin-orbit split CB and the topmost VB at the K, K' points, with the brighter lower energy emission line being spin-forbidden and hence phonon activated. An emerging emission feature at low temperature is assigned to defect bound states.

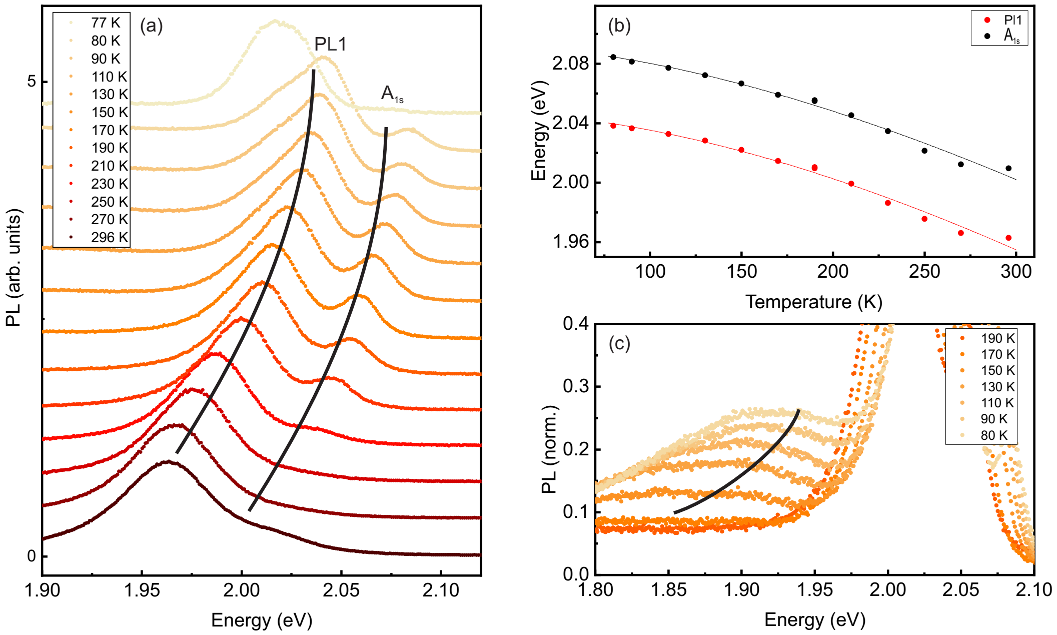

Figure S3. Temperature dependent PL spectra of a WS_2 ML. (a) PL spectra within a 77 - 300K temperature range with two dominating peaks $P1$ and A_{1S}. Solid black lines indicate peak positions and serve as a guide to the eye. (b) Peak positions of $P1$ and A_{1S} plotted against temperature. Solid lines represent an empirical Varshni fit [1]. Fit parameters are summarized in S1. (c) Emerging low temperature feature that is assigned to defect activated emission. Solid black line is a guide to the eye.

Table S1. Fit values of semi-empirical Varshni fits of temperature dependent optical bandgaps extracted from PL peak positions. Fit is performed employing $E_g(T) = E_g(0) - \frac{\alpha T^2}{T+\beta}$.

Peak	Parameter	Value	Uncertainty	unit
P1	E_g	2.048	0.002	eV
	α	1.229E-3	1.171E-3	eV/K
	β	881	1149	K
A_{1s}	E_g	2.093	0.004	eV
	α	1.021E-3	2.001E-3	eV/K
	β	713	789.1	K

2. Spectroscopic Imaging Ellipsometry

Modelling of the experimental Spectroscopic Imaging Ellipsometry (SIE) spectra was carried out employing a suitable multilayer model and regression analysis as described in detail for instance in Ref. [2]. In a first step, the dielectric function of WS_2 on a bare glass substrate is measured and modelled (Fig. S4a)-c)) to determine the numeric description required to describe the dielectric function of WS_2 ML. Consequently, the structure was extended to a layerstack including also a PE layer, changing the parameters of the WS_2 ML. Finally, a gate voltage was applied to the device and the voltage induced changes were modeled with starting parameters given from the model of the unbiased SIE spectra.

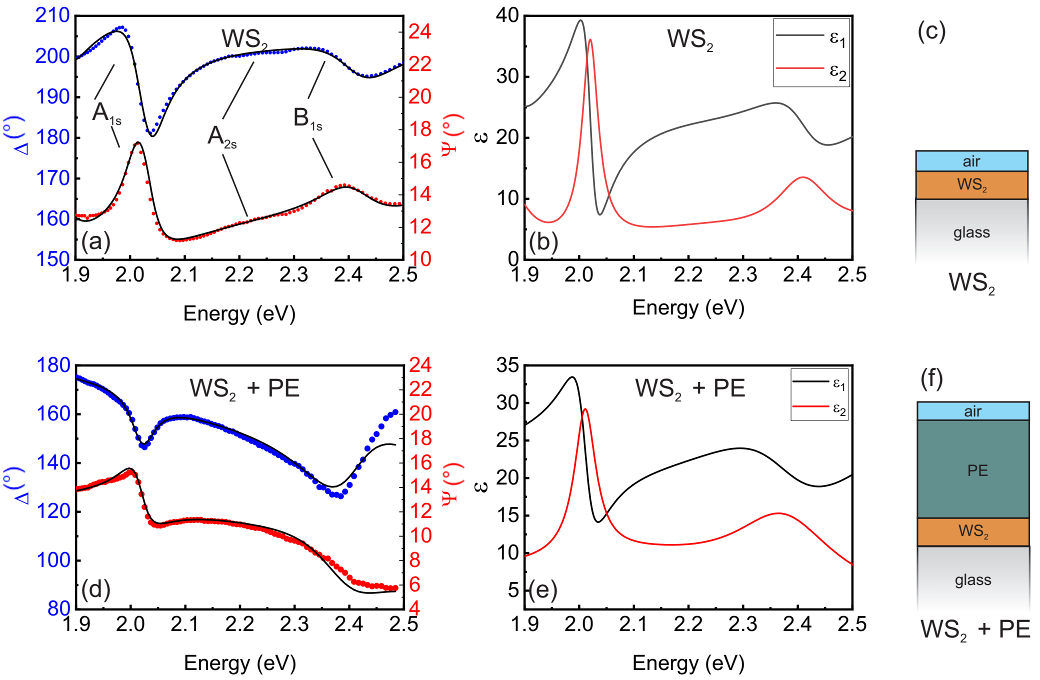

Figure S4. Ellipsometry spectra of different layer structures. (a) Δ and ψ spectra of a WS_2 ML deposited on glass. Solid black lines represent modeled data. Excitonic features are indicated with lines. (b) Extracted dielectric function of the WS_2 ML. (c) Illustration of the layer structure for the bare WS_2 case (d) Δ and ψ spectra of a WS_2 ML coated with PE deposited on glass. Solid black lines represent modeled data. (e) Extracted dielectric function of a WS_2 ML coated with PE. (f) Illustration of the layer structure for the encapsulated WS_2 ML.

3. Reproducibility

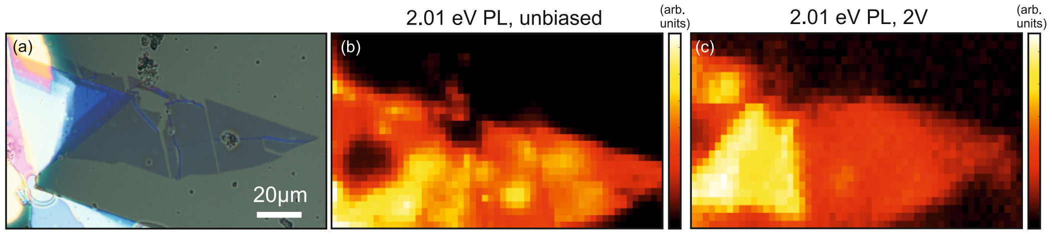

Figure S5. Optical micrograph and PL maps of a WS_2 ML. (a) Image of a WS_2 ML contacted to a gold contact and coated with a 350nm PE layer. (b) and (c) μ-PL maps of the ML shown in (a) for the unbiased case [(b)] and for an applied gate voltage of 2V [(c)]. The PL emission of disrupted part of the WS_2 ML is not changed when a gate voltage is applied.

Overall, more than 8 samples have been prepared and studied and some of the samples have been intensely measured with several gate cycles. To rule out a possible effect of the gold contact on the optical response of the WS_2 ML, μ-PL maps have been carried out at different gate voltages. No dependence from the distance of the PL signal on the distance to the gold contact was observed (Fig. S5). The disrupted part of the WS_2 ML shows no dependence on the gate voltage, as its PL is not enhanced under an applied gate voltage of 2V (Fig. S5c)). The overall behaviour, particularly the change of the PL emission in the doped regime is very well reproducible and very similar for different samples as shown on example of gate dependent PL measurements for both, gate- up and down sweep, carried out on two different WS_2 ML FET structures (Fig. S6). For better comparison and due to the established gate-hysteresis, the gate voltage values at which E_F crosses the CB or VB, respectively, have been shifted to zero. The voltage values at which E_F touches the CB or VB band edges are marked by local maxima in the integrated PL intensities.

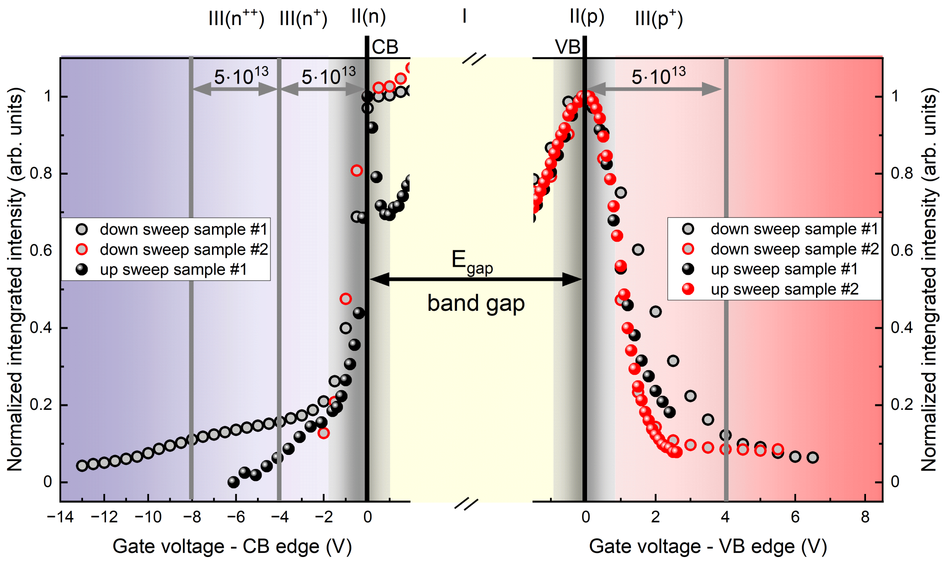

Figure S6. Comparison of the gate voltage dependence of the integrated intensities from two different WS_2 ML FET structures for gate- up and down sweeps. For better comparison, the gate voltage values are shifted to zero for the values at which E_F crossed CB and VB edges, respectively, identified by the intensity maxima at CB and VB band side. As explained in the main text, the used FET-structure is not suitable to quantify the band gap energy E_{gap}, but it can be determined qualitatively in the experiments. For the situation, when E_F is in the VB or VB edge, the well known plate capacitor model is applicable as described in the main text allowing for the estimation of the gate induced charge carrier densities. The corresponding doping regime for intrinsic [I], slightly n- or p-doped [II(n/p)], highly n- or p-doped [II(n^+/p^+)] and extremely highly doped [II(n^++/p^++) regimes are indicated. The measurements proof the reproducible as well as universality of the experimental signatures with minor deviations between different samples.

References

1. Varshni, Y. Temperature dependence of the energy gap in semiconductors. *Physica* **1967**, *34*, 149–154. https://doi.org/https://doi.org/10.1016/0031-8914(67)90062-6.
2. Funke, S.; Miller, B.; Parzinger, E.; Thiesen, P.; Holleitner, A.; Wurstbauer, U. Imaging spectroscopic ellipsometry of MoS2. *Journal of Physics: Condensed Matter* **2016**, *28*, 385301.
